# Isokinetic Testing After Anterior Cruciate Ligament Injury Showed a Greater Hamstrings/Quadriceps Ratio at 240°/S Over 6 Months From Injury but No Difference of Limb Symmetry Index

**DOI:** 10.1016/j.asmr.2024.101063

**Published:** 2024-12-12

**Authors:** Guillaume Mesnard, Gaspard Fournier, Nicolas Cance, Robert A. Magnussen, Sébastien Lustig, Elvire Servien

**Affiliations:** a Orthopaedics Surgery and Sports Medicine Department, FIFA Medical Center of Excellence, Croix-Rousse Hospital, Lyon University Hospital, Lyon, France; b Department of Orthopaedics, The Ohio State University, Columbus, Ohio, U.S.A.; c OSU Sports Medicine Research Institute, The Ohio State University, Columbus, Ohio, U.S.A.; d Univ Lyon, Claude Bernard Lyon 1 University, IFSTTAR, LBMC UMR_T9406, F69622, Lyon, France; e LIBM - EA 7424, Interuniversity Laboratory of Biology of Mobility, Claude Bernard Lyon 1 University, Lyon, France

## Abstract

**Purpose:**

To assess preoperative quadriceps and hamstring strength at various time points after anterior cruciate ligament (ACL) injury but before ACL reconstruction.

**Methods:**

Patients who underwent isokinetic muscle strength testing before planned ACL reconstruction were included. Patients were placed in 1 of the following 3 groups on the basis of time from injury to testing: <3 months, 3 to 6 months, and 6 to 12 months. Among these 3 groups, hamstring and quadriceps limb symmetry index (LSI) and hamstring/quadriceps (H/Q) ratios were compared. A total of 100 patients were included (<3 months [n = 55]; 3-6 months [n = 31]; and 6-12 months [n = 14]).

**Results:**

There were no significant differences between patients in the 3 groups in regards to age, body mass index, or flexion range of motion, but there was a greater proportion of female patients in the 6 to 12 month group than the other groups. No significant differences in quadriceps or hamstring strength were noted among the 3 groups. H/Q ratio was significantly greater in the 6 to 12 month group than the less than 3 months and 3 to 6 months groups, with concentric testing at 240°/s. No correlation was found between patient sex and LSI or H/Q ratios.

**Conclusions:**

Patients who underwent isokinetic muscle strength testing 6 to 12 months after ACL injury had a greater H/Q ratio at 240°/s than those who were testing within 6 months of injury. No differences in hamstring or quadriceps LSI were noted on the basis of time.

**Clinical Relevance:**

An understanding of the factors that influence preoperative isokinetic muscle strength testing (including time from injury to surgery) is important, given associations noted between preoperative strength and postoperative strength recovery after ACL reconstruction.

Anterior cruciate ligament (ACL) rupture is a common injury in athletic populations.^1^, ^2^, ^3^ The management of patients with ACL injuries is increasingly multidisciplinary, with the goal of a quick and safe return to sport.^4^ The evaluation of postoperative muscle strength recovery is an integral part of the rehabilitation process. Numerous studies have shown the importance of recovery of muscle strength for successful return to sport^5^^,^^6^ to avoid risk of rerupture.^7^

Isokinetic muscle strength testing frequently is performed as an objective measurement of muscle recovery during rehabilitation.^8^^,^^9^ Although strength recovery is an important focus throughout postoperative rehabilitation, muscle strength loss in the affected limb begins as soon as the injury occurs. In order to maximize early strength restoration postoperatively, preoperative muscle recovery also must be considered.^10^ Many authors have evaluated the evolution of the postoperative strength deficits and noted an association with preoperative strength.^11^, ^12^, ^13^

The limb symmetry index (LSI) is an objective parameter of muscle strength and is important in ACL rehabilitation. It involves comparing the performance of the affected leg with the unaffected leg. The LSI can be assessed using a number of parameters^14^, ^15^, ^16^, ^17^ but isokinetic testing remains the gold standard. The hamstrings/quadriceps (H/Q) ratio reflects the overall balance of the limb between knee extensors and flexors. This balance is essential to reduce the risk of further injury when returning to sport.^18^

Despite the known impact of preoperative strength on recovery after surgery, there is limited literature exploring factors that could influence preoperative strength. Of particular interest is the relationship between time from injury and preoperative hamstring and quadriceps strength. The purpose of this study is to assess preoperative quadriceps and hamstring strength at various time points after ACL injury but before ACL reconstruction. We hypothesized that less side-to-side strength deficit would be seen at longer time points after ACL injury.

## Methods

### Population

A retrospective study was performed on the basis of prospectively collected data. All procedures were performed following institutional board approval and in accordance with the ethical standards of the 1964 Helsinki declaration and its later amendments. Patients who underwent ACLR between July 2020 and April 2022 at 1 academic center were eligible for inclusion in the study if that had undergone preoperative isokinetic muscle strength testing within 1 year of their ACL injury. Patients were excluded from the study if they did not undergo preoperative isokinetic muscle strength testing or the time from ACL injury to isokinetic muscle strength testing was greater than 1 year, to rule out old ruptures of the ACL. During this period, preoperative isokinetic muscle strength testing was performed on all patients undergoing ACL reconstruction except for those who could not undergo testing because of pain or the presence of multiligament injuries, bucket-handle meniscus tears, or other associated lesions. Every patient underwent an isolated isokinetic muscle strength testing. Rehabilitation before surgery was mandatory only in cases of deficit of range of motion.

Data collection and analysis were carried out in accordance with MR004 Reference Methodology from the Commission Nationale de l'Informatique et des Libertés (ref. 2226075), obtained on April 19, 2022. The study was registered and filed on the Health Data Hub website. Informed consent was obtained from all individual participants included in the study.

Patient demographic data were collected including age, sex, body mass index (BMI), Tegner activity scale, and knee range of motion at the time of isokinetic muscle strength testing. Patients were divided into 3 groups on the basis of time from ACL injury to their isokinetic muscle strength testing (<3 months, 3-<6 months, and >6-12 months).

### Isokinetic Muscle Strength Testing

Isokinetic muscle strength testing was performed using the Contrex (Physiomed Elektromedizin AG, Schnaittach, Germany) machine. A standardized protocol was used, beginning with 15 minute warm-up on a stationary bike. For each testing condition, patients performed several submaximal trials for familiarization with the procedure, then maximal effort testing on the uninjured side followed by the injured side. Concentric testing of the hamstring and quadriceps was performed first, with 3 repetitions at 60°/s, then 3 repetitions at 240°/s. Eccentric tests of the hamstrings were then performed with 3 repetitions at 30°/s. Tests were done with a 70° range of motion (20°-90°).

On the basis of the greater of the 3 repetitions, LSI comparing the injured side to the uninjured side (percentage) was calculated using peak torque (in Newton-meters), for the quadriceps and hamstring muscle groups. For each concentric condition, the H/Q ratio was calculated.

### Statistical Analysis

Continuous variables were compared among the 3 time-based groups using Kruskal-Wallis test, and Bonferroni post hoc test was used in case of significant difference. Categorical variables were compared among the 3 groups using Fisher exact tests. Isokinetic muscle strength testing results were compared on the basis of sex using Mann-Whitney *U* tests. All analyses were performed using XLSTAT Software (V2021.1; Addinsoft, Paris, France), with *P* < .05 considered statistically significant.

## Results

**Fig 1:**
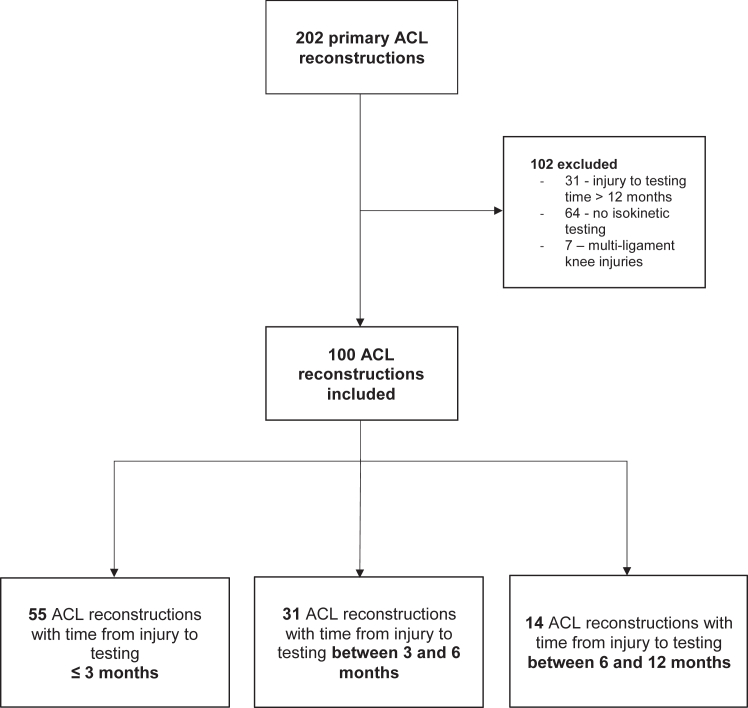
Flowchart. (ACL, anterior cruciate ligament.)

In the study period, 202 patients underwent primary ACL reconstruction in the department. After exclusion of 31 patients with a history of an ACL tear greater than 12 months before presentation, 7 patients with multiligament knee injuries, and 64 patients who did not undergo preoperative isokinetic muscle strength testing, 100 patients were eligible and included in study (Fig 1).

Patients were grouped on the basis of time from ACL injury to isokinetic muscle strength testing, yielding 55 patients who underwent testing with 3 months of ACL injury, 31 patients who underwent testing between 3 and 6 months after ACL injury, and 14 patients who underwent isokinetic muscle strength testing between 6 and 12 months after ACL injury. The overall study population consisted of 69 male (69%) and 31 female (31%) patients with a mean age of 29.1 years and a mean BMI of 24.3. The mean overall time from injury to isokinetic muscle strength testing was 81 days. There were no significant differences between patients in the 3 groups in regards to age, BMI, Tegner activity scale, or flexion range of motion, but there was a greater proportion of female patients in the 6 to 12 months group when compared with the other groups (Table 1). In total, 31 patients did not undergo rehabilitation before surgery (14 patients in the <3 months group, 13 in the 3-6 months group, and 4 in the >6 months group)

**Table 1:** Demographics and Range of Motion

Demographics	Time From Injury to Surgery			Significance
	<3 Months (n = 55)	3 to 6 Months (n = 31)	6 to 12 Months (n = 14)	
Sex				.02
Female	18 (33%)	6 (19%)	7 (50%)	
Male	37 (67%)	25 (81%)	7 (50%)	
Age, yr, mean ± SD	27.6 ± 10.4	31.4 ± 11.9	29.6 ± 12.1	.384
BMI, mean ± SD	24.6 ± 4.9	24.7 ± 3.5	24.7 ± 10.4	.674
Days from injury to surgery, mean ± SD	56.2 ± 21.3	128.1 ± 28.7	272.1 ± 70.1	<.001
Tegner activity scale	6.8 ± 1.8	6.5 ± 1.8	6.1 ± 1.3	.361
Range of motion				
Flexion, ° mean ± SD	131 ± 15	134 ± 11	137 ± 8	.406
Flexion contracture >5°	4 (7.3%)	7 (22.6%)	0 (0%)	.060

SD, standard deviation.

### Isokinetic Muscle Strength Testing

The mean overall quadriceps LSI was 81.2 ± 23.6% at 60°/s and 83.6 ± 22.1% at 240°/s. The mean overall hamstring LSI was 85.3 ± 20.9% at 60°/s, 90.1 ± 19.3% at 240°/s, and 81.9 ± 19.6% with eccentric testing at 30°/s. No significant difference in quadriceps or hamstring LSI was identified on the basis of the time from injury to isokinetic muscle strength testing (Table 2). The hamstring/quadriceps ratio was noted to be significantly greater in the 6 to 12 months group when compared with the <3 months and 3 to 6 months groups with concentric testing at 240°/s (*P* = .05 and *P* = .006, respectively, at Bonferroni test; Bonferroni-corrected significance level: .0167). No difference on the basis of time from injury to testing were noted under other testing conditions (Table 2). No differences in isokinetic muscle strength testing were found on the basis of patient sex (Table 3).

**Table 2:** Isokinetic Muscle Strength Testing LSI (Using Peak Torque) by Time

	Time From Injury to Surgery			Significance
	<3 Months (n = 55)	3 to 6 Months (n = 31)	6 to 12 Months (n = 14)	
Concentric tests at 240°/s, mean ± SD				
Hamstring	89.0 ± 18.2%	90.5 ± 22.2%	91.9 ± 19.6%	.90
Quadriceps	84.5 ± 18.9%	81.8 ± 27.7%	84.6 ± 20.8%	.99
Concentric tests at 60°/s, mean ± SD				
Hamstring	81.6 ± 21.4%	87.8 ± 19.1%	93.7 ± 21.3%	.15
Quadriceps	81.9 ± 18.8%	81.4 ± 29.0%	85.5 ± 21.0%	.92
Eccentric tests at 30°/s, mean ± SD				
Hamstring	77.7 ± 19.8%	86.0 ± 20.6%	89.3 ± 19.3%	.12
Hamstring/quadriceps ratio				
Concentric at 240°/s	75.8 ± 16.7%	76.8 ± 42.7%	96.6 ± 48.4%	.020
Concentric at 60°/s	62.7 ± 15.0%	68.6 ± 32.1%	84.3 ± 56.7%	.33

LSI, limb symmetry index; SD, standard deviation.

**Table 3:** Isokinetic Muscle Strength Testing LSI (Using Peak Torque) by Sex

	Male (n = 69)	Female (n = 31)	Significance
Concentric tests at 240°/s, mean ± SD			
Hamstring	88.8 ± 18.3%	93.0 ± 21.2%	.51
Quadriceps	83.8 ± 23.0%	84.0 ± 20.4%	.96
Concentric tests at 60°/s, mean ± SD			
Hamstring	85.9 ± 20.6%	83.9 ± 21.7%	.82
Quadriceps	82.0 ± 23.0%	81.3 ± 21.6%	.66
Eccentric tests at 30°/s, mean ± SD			
Hamstring	82.6 ± 20.0%	80.3 ± 19.8%	.72
Hamstring/quadriceps ratio			
Concentric at 240°/s	80.6 ± 37.6%	76.1 ± 16.5%	.947
Concentric at 60°/s	70.0 ± 35.1%	62.8 ± 12.3%	.917

LSI, limb symmetry index; SD, standard deviation.

## Discussion

The most important finding of this study is that there was no significant difference in quadriceps or hamstring isokinetic LSI testing on the basis of time from ACL injury to isokinetic muscle strength testing. However; a significantly greater H/Q ratio was noted in the 6 to 12 month group compared with the other 2 groups with concentric testing at 240°/s. This finding is in contrast to our hypothesis that less side-to-side strength deficit would be seen at longer time points after ACL injury.

A similar lack of clear correlation between lower-extremity strength and time from injury has been noted by other authors. Wojtys and Huston^19^ studied lower-extremity strength in 100 patients with ACL injuries who were treated nonoperatively. Patients were grouped on the basis of the time from injury to testing (up to 18 months). These patients were compared with healthy controls, and it was noted that a substantial portion of patients in each group showed ongoing weakness compared with control patients, particularly in the quadriceps. However, patients with the best muscle strength in all group were similar to control patients.

A clear understanding of factors that influence preoperative isokinetic muscle strength testing (including time from injury to surgery) is important, given associations noted between preoperative strength and postoperative strength recovery. Riesterer et al.^20^ in 2017, studied 80 patients who underwent primary hamstring ACL reconstruction with preoperative and 6-month postoperative isokinetic testing. They noted that postoperative strength was strongly correlated with preoperative strength and confirmed the adverse impact of low postoperative strength on knee function.

In the current study, there are signs that recovery of the quadriceps and hamstring muscle groups after ACL injury does not follow the same course. Nonsignificant differences were seen in recovery of hamstring strength with increasing time from injury, although the differences did not reach statistical significance. This was also reflected in evolution of H/Q ratios over time, with a significant increase in the H/Q ratio at high speeds with time. Strictly time-based criteria to allow for strength recovery may not be recommended on the time magnitudes provided in the current study. It is possible that with preoperative rehabilitation/intervention, this could be improved, or shorter time frames may show a significant difference.

This differential recovery between quadriceps and hamstring strength after ACL injury has been noted by other authors. Tsepis et al.^21^ noted this difference after ACL injury in 36 amateur soccer players, with total recovery of hamstring muscle strength but persistent quadriceps weakness at 1 year after injury. In the postoperative setting, Olivier et al.^22^ found complete recovery of hamstring strength by 4 months postoperatively but persistent quadriceps weakness at that time point.

Given the negative impact of postoperative quadriceps strength on outcomes and the correlation between preoperative and postoperative strength, numerous authors have evaluated the effectiveness of preoperative rehabilitation programs. More intensive preoperative rehabilitation resulted in better functional results in the short^23^ and longer term^24^^,^^25^ postoperatively (up to 2 years). These correlations have also led to a focus on the goal of restoring quadriceps during post-operative rehabilitation.^26^, ^27^, ^28^

### Limitations

There are several limitations of the current study. The largest limitation is the cross-sectional study design. Because isokinetic muscle strength testing was only performed at one time point on each subject, it is not possible to study the evaluation of deficits over time in individual patients. Second, the greater proportion of female patients could interfere with the generalizability of the results. The study likely remains underpowered to detect strength differences between groups because of the high variability in isokinetic muscle strength values. Further, the preoperative rehabilitation protocol was not standardized, leading to a heterogeneous population. Finally, there may be other differences between the patients tested at different time points that are not known or controlled for. These differences may confound the results.

## Conclusions

Patients who underwent isokinetic muscle strength testing 6 to 12 months after ACL injury had a greater H/Q ratio at 240°/s than those who were testing within 6 months of injury. No differences in hamstring or quadriceps LSI were noted on the basis of time.

## Disclosures

The authors declare the following financial interests/personal relationships which may be considered as potential competing interests: R.M. reports research funding from 10.13039/100031014Vericel and 10.13039/100009026Smith & Nephew and Editorial Board for the *Journal of the American Academy of Orthopaedic Surgeons* and *Orthopaedic Journal of Sports Medicine*. E.S. reports consultant for Corin. S.L. reports consultant for Stryker, Smith & Nephew, Heraeus, and DePuy Synthes; institutional research support from Lepine and Amplitude; and Editorial Board for the *Journal of Bone and Joint Surgery (Am*). All other authors (G.M. G.F., N.C.) declare that they have no known competing financial interests or personal relationships that could have appeared to influence the work reported in this paper.
